# Flavivirus genome detected in *Mus *and *Apodemus *species sampled in illegal waste sites

**DOI:** 10.1186/1756-3305-7-S1-P13

**Published:** 2014-04-01

**Authors:** D Duh, E Varljen-Buzan, U Kunej, S Hasic

**Affiliations:** 1 Science and Research Centre, University of Primorska, Koper - Capodistria, Slovenia; 2 Faculty of Mathematics, Natural Sciences and Information Technologies University of Primorska, Koper - Capodistria, Slovenia

## 

Tick-borne encephalitis virus (TBEV), a zoonotic flavivirus that occurs on the Eurasian continent and causes tick-borne encephalitis (TBE) in humans, is considered medically the most important arthropod vector transmitted virus in Europe. As such, TBEV is of course not a neglected pathogen. However, the knowledge about TBEV could importantly contribute to the research of other neglected vector borne pathogens. In nature, TBEV is transmitted from tick to tick via vertebrates in a process named co-feeding which is independent of a systemic viraemia in vertebrates. Therefore the ticks are the reservoirs as well as the vectors for TBEV, while the vertebrate is considered the transient bridge in transmission and maintenance of TBEV.

Slovenia is an endemic country for TBEV with approximately 300 cases reported annually and incidence around 14 per 100,000 inhabitants. It was shown, that TBEV in Slovenia is maintained in tick-rodent-deer cycle. TBEV viraemic rodents of *Myodes *and *Apodemus *species were described previously in Slovenia. We were interested whether TBEV could be present in *Mus *species which share a living habitat with *Apodemus *mice in illegal waste sites. Thus we collected 83 samples of *Mus *and *Apodemus *species from different dump sites in Slovenia and Croatia. The presence of TBEV was determined using molecular techniques. Total RNA was extracted from spleen samples using RTP Pathogen Kit (Invitek-Stratec) and tested with real time RT-PCR specific for TBEV-Eur subtype. All samples were negative which could be explained by the fact that testing area was in the part of Slovenia with low incidence of TBE. However, distinctive samples were also tested with universal primers for amplification of flavivirus genome. Six amplicons of correct size were seen on agarose gel and are in the process of sequencing to reveal the identity of detected flavivirus.

